# Diagnosis and mortality in *47,XYY *persons: a registry study

**DOI:** 10.1186/1750-1172-5-15

**Published:** 2010-05-29

**Authors:** Kirstine Stochholm, Svend Juul, Claus H Gravholt

**Affiliations:** 1 Department of Internal Medicine and Endocrinology, Aarhus Sygehus, Aarhus University Hospital, Norrebrogade 44, 8000 Aarhus C, Denmark; 2 Department of Epidemiology, School of Public Health, Aarhus University, Denmark

## Abstract

**Background:**

Sex chromosomal abnormalities are relatively common, yet many aspects of these syndromes remain unexplored. For instance epidemiological data in *47,XYY *persons are still limited.

**Methods:**

Using a national Danish registry, we identified 208 persons with *47,XYY *or a compatible karyotype, whereof 36 were deceased; all were diagnosed from 1968 to 2008. For further analyses, we identified age matched controls from the male background population (n = 20,078) in Statistics Denmark. We report nationwide prevalence data, data regarding age at diagnosis, as well as total and cause specific mortality data in these persons.

**Results:**

The average prevalence was 14.2 47,XYY persons per 100,000, which is reduced compared to the expected 98 per 100,000. Their median age at diagnosis was 17.1 years. We found a significantly decreased lifespan from 77.9 years (controls) to 67.5 years (*47,XYY *persons). Total mortality was significantly increased compared to controls, with a hazard ratio of 3.6 (2.6-5.1). Dividing the causes of deaths according to the International Classification of Diseases, we identified an increased hazard ratio in all informative chapters, with a significantly increased ratio in cancer, pulmonary, neurological and unspecified diseases, and trauma.

**Conclusion:**

We here present national epidemiological data regarding *47,XYY *syndrome, including prevalence and mortality data, showing a significantly delay to diagnosis, reduced life expectancy and an increased total and cause specific mortality.

## Background

One of the first descriptions of *47,XYY *is from 1965 by Jacobs et al [1]. Here a chromosome survey of male patients at the State Hospital in Carstairs was conducted. The hypothesis being that *47,XYY *was particularly frequent among inmates in penal institutions. Later, other studies took place in hospitals among consecutively born babies [2-7] using techniques enabling the identification of extra Y chromosome material. These studies identified highly variable number of *47,XYY *persons, ranging in liveborn from 26 per 100,000 [2] to 375 per 100,000 [4]. We calculated the prevalence of *47,XYY *at birth by pooling data from the surveys in consecutively liveborn babies in various countries and estimated the prevalence of *47,XYY *to 98 per 100,000 (95% CI: 73-129) (51 *47,XYY *persons out of 52,004 liveborn boys). A few earlier chromosomal studies only used identification of Barr bodies, for instance from smears from the oral mucosa, hence no Y chromosome defects were identified [8,9]. By comparison, it is estimated that sex chromosomal abnormalities occur in 1 per 400 births [10].

Only limited data regarding age at diagnosis in *47,XYY *syndrome in a relatively unselected population are available [11]. To date, not much is generally known regarding clinical phenotype of individuals with *47,XYY*, except tall stature [12], possibly due to the expression of three copies of the short statue homeobox-containing gene (SHOX), which is located on the distal ends of Xp and Yp in the pseudoautosomal region 1 (PAR1) [13].

One British study [14] identified a significantly increased mortality in total and in diseases of the respiratory system in a *47,XYY *population compared to the background population. However, to our knowledge nothing is known concerning prevalence and mortality in a nationwide identified *47,XYY *population. We therefore undertook the present Danish nationwide study, investigating age at diagnosis, prevalence and mortality in a cohort of 47, XYY persons and comparing this to a large background population.

### Identification of 47,XYY persons

The Danish Cytogenetic Central Registry was founded in 1967 and contains all national data regarding the diagnoses of chromosomal anomalies, including information from the years before 1967. The first diagnosis of 47,XYY in Denmark was in 1965. We retrieved the identification (ID) number of all Danish men ever diagnosed with *47,XYY *in Denmark, referred to here as index-persons. As an index-person we accepted variants of *47,XYY *including mosaics (*46,XY*/*47,XYY*). Due to their more severe phenotype *48,XXYY *males (n = 23) and *48,XYYY *males (n = 1) were not included as well as *47,XYY *males with an autosomal aneuploidy (n = 1), as their sex chromosomal aneuploidy was considered of minor importance. Hereby 208 index-persons were identified, for details see table 1. It is important to emphasize that no information regarding phenotype or reasons for which the chromosomal analyses were performed were included in the registry. In 1968 and onwards, one unique ID-number was allocated to every living Dane. Date of birth and gender can easily be identified using the ID-number. The ID-numbers as well as date of diagnosis were retrieved from the Danish Cytogenetic Central Registry in March 2009.

**Table 1:** Details regarding the karyotypes

Subgroup	Specific karyotype	Number
*47,XYY*	*47,XYY* *47,XYY,inv(9)* Subtotal	175 2 177

*46,XY/47,XYY*	*46,XY/47,XYY* *45,X/46,XY/47,XYY* *45,X/47,XYY* Subtotal	20 1 1 22

Others	*47,XY,?YQ-* *47,XYY/48,XXYY* *47,XY,r(Y)(P11Q11)* *45,X/46,X,idic(Y)(P11.3)/47,X,idic(Y)(P11.3)* *47,XYY,inv(5)(P13.3P15.33)* *46,XY,t(9;19)(Q21;Q13)/47,XYY,t(9;19)(Q21;Q13)* *46,XY/47,XY,+I(YQ) 45,X/45,Y/46,XY/47,XYY* Subtotal	2 1 1 1 1 1 1 1 9

Total		208

Details regarding all men diagnosed with a karyotype compatible with *47,XYY *in Denmark during 1965 to 2008.

### Identification of controls

Statistics Denmark was founded in 1850, and contains numerous statistics regarding Denmark and Danes. Using the ID-number, Statistics Denmark http://www.dst.dk identified for each index-person up to 100 controls from the male background population, matched on age (year and month of birth). All controls were alive and living in Denmark on the date their index-person was diagnosed with 47,XYY. For two index-persons the matching was not undertaken for unknown reasons, and mortality data are therefore on 206 *47,XYY *persons. Furthermore, we retrieved data regarding date of emigration, date of death, and up to three causes of death on these men. None were lost to follow-up.

### Mortality

All controls were alive and registered in Denmark on the day the index-person was diagnosed with *47,XYY*. Mortality data was updated with 31^st ^of December 2008 being the last date of death registered, whereas causes of death were updated until 31^st ^of December 2006. Thus, all who were deceased in 2007 and 2008 were registered with date of death, but without registration of causes of death.

The causes of death were given in International Classification of Diseases (ICD) 8^th ^edition until 1993, and in ICD-10 and onwards. We translated ICD-8 diagnoses to ICD-10, and divided all deaths into 19 chapters according to ICD-10 for analysis of cause-specific mortality. Cause-specific mortality hazard ratios (HR) were calculated for each of the 19 chapters in ICD-10, as well as in total.

## Statistics

The calculation of the expected prevalence was performed by adding all 47,XYY persons or variants identified in screening studies as well as the number of liveborn boys investigated. The confidence intervals were calculated using the poisson distribution.

To compare median age at diagnosis, median date of birth and median date of diagnosis in the three subgroups, we used the Kruskal-Wallis test. The time trend in age at diagnosis was analyzed using linear regression.

The prevalence was calculated as number of diagnoses per 100 000 liveborn boys in the background population per year of diagnosis. Confidence intervals were estimated using an approximation to the Poisson distribution. The specific numbers of liveborn boys in the background population were obtained using Statistics Denmark. To identify changes in prevalence per year of diagnosis we used Poisson regression. The average prevalence was calculated as average number of incident *47,XYY *persons during 1970-2008 divided by the average number of liveborn boys in the background population during the same period. This study period was applied to ensure enough run-in time in the Danish Cytogenetic Registry from time of establishment.

Mortality was described with Kaplan-Meier survival estimates constructed using date of birth as entry. Date of emigration, date of death or 31^st ^of December 2008, whichever came first, were used as date of exit. For comparison log-rank analysis was applied.

HRs were calculated using Cox regression analysis with stratification, using each person and his matched controls as a stratum. Hereby, comparisons were adjusted for age and calendar time, and calculation of expected number of deaths was possible. Time at risk was calculated as time from date of diagnosis until date of emigration, date of death or 31^st ^of December 2008, whichever came first, this applied to both index-persons and controls. All results are shown with 95% confidence intervals, or with range if relevant, and p < 0.05 was considered statistically significant. We made no formal corrections for multiple comparisons. We used Stata 10.0 (Stata Corp. College Station,TX, USA) for all calculations.

## Results

**Figure 1:**
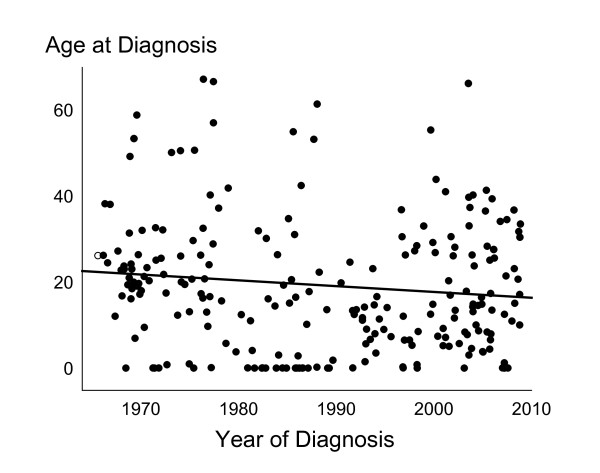
**Age at diagnosis in all males diagnosed in Denmark with *47,XYY *during 1965 to 2008**.

**Figure 2:**
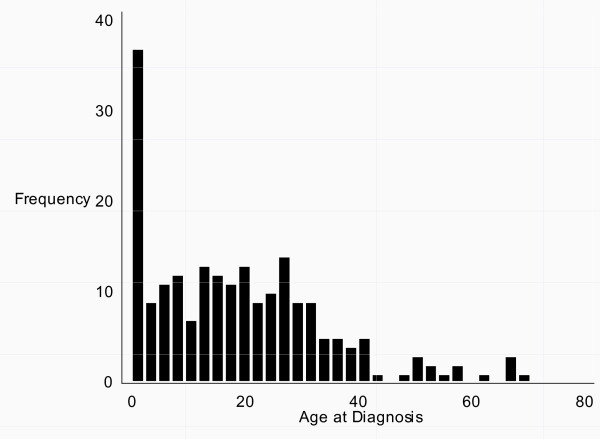
**Age at diagnosis in all males diagnosed in Denmark with *47,XYY *during 1965 to 2008**.

In the Danish Cytogenetic Central Registry we identified 208 males diagnosed between 1965 and 2008 with *47,XYY *or a compatible karyotype and divided them into three subgroups (Table 1). Age at diagnosis, year of birth and year of diagnosis is seen in Table 2. The vast majority (n = 177) were non-mosaic *47,XYY*. In all three subgroups age at diagnosis had a wide range, spanning more than 65 years. The subgroup of "Others" (n = 9) were both significantly older than the other two subgroups at diagnosis (both p < 0.05), as well as born significantly earlier in the study period (both p < 0.05). In the total cohort, age at diagnosis significantly decreased during the study period (p < 0.05, Figure 1), as in the mosaic subgroup (p < 0.05). The distribution of age at diagnosis is seen in Figure 2. Twenty-five percent were diagnosed within the age of 5.9 years, 50% with the age of 17.1 years, and 75% within the age of 28.0 years.

**Table 2:** Details regarding *47,XYY *persons.

Karyotype	Number of persons	Median age at diagnosis (range)	Median year of birth (range)	Median year of diagnosis (range)
*47,XYY*	177	16.9 (0.0-67.1)	1971.9 (1909.4-2007.3)	1988.1 (1965.7-2008.9)

*46,XY/47,XYY*	22	6.9 (0.0-70.7)	1983.9 (1899.8-2007.6)	1996.3 (1968.9-2007.6)

Others	9	28.8 ^1)^ (0.0-66.5)	1948.6 (1910.9-1984.5)	1977.5 ^1)^ (1969.1-2006.2)

Total	208	17.1 (0.0-70.7)	1972.4 (1899.8-2007.6)	1988.2 (1965.7-2008.9)

Details regarding all men diagnosed with a karyotype compatible with *47,XYY *in Denmark between 1965 to 2008.

^1)^p-value < 0.05 compared to both of the other subgroups.

**Figure 3:**
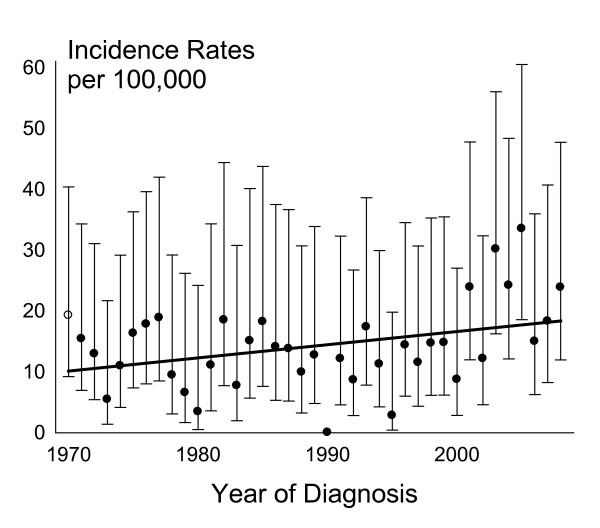
**Prevalence rate of *47,XYY *males in Denmark during 1970 to 2008**. The number of diagnosed *47,XYY *males in Denmark during 1970-2008 per 100,000 liveborn boys per year of diagnosis.

During 1970-2008 we identified 180 *47,XYY *persons, corresponding to an average of 4.6 persons yearly. With an average of 32 794 liveborn boys in Denmark we thus identified 14.1 *47,XYY *persons per 100 000 liveborn. Prevalence per 100 000 liveborn boys in the background population were stable during the study period (p = 0.96) (Figure 3), however there seemed to be an increase from 1995 until 2008.

**Figure 4:**
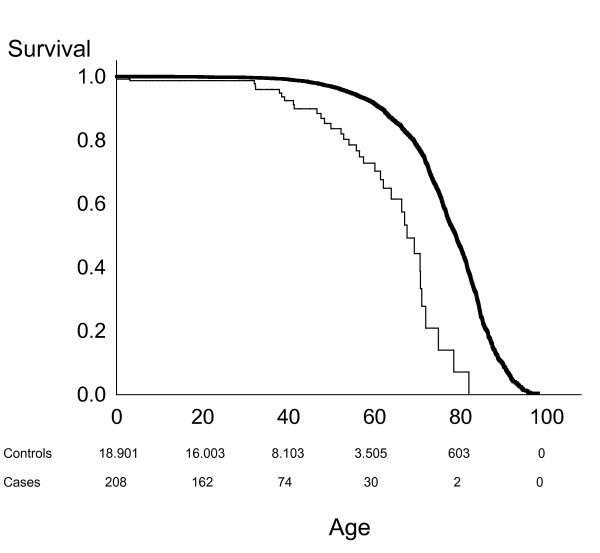
**Kaplan-Meier survival graphs in *47,XYY *compared to an age-matched male background population**. Time at risk was calculated from date of birth until date of censoring (see Materials and Methods for details). Solid line controls, and thin line persons. Survival is significantly lower in *47,XYY *persons, log-rank p < 0.0001. Number of persons and controls are indicated below the figure.

In Statistics Denmark we identified 20 078 matched controls; a minimum of 82 controls were identified per index-person. During the study period 1 895 controls and 36 index-persons died, whereof ten deaths were expected. Twenty-eight index-persons died in the 47,XYY subgroup, four in the mosaic subgroup and four in the subgroup of others. Mortality was significantly increased in *47,XYY *persons in total (p < 0.0001) (Figure 4) and in all three subgroups (all p < 0.0005). Time at risk, corresponding to time from date of diagnosis to date of exit, was 3 373 years in the index-persons and 373 946 years in the controls. The median age of survival was 77.9 years for controls and 67.5 years for *47,XYY *persons, corresponding to a loss of median lifespan of 10.3 years.

**Figure 5:**
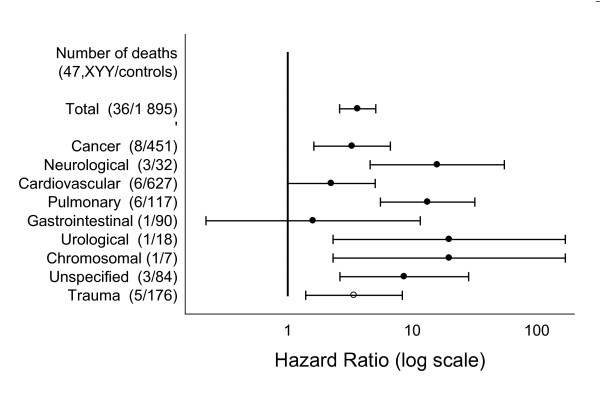
**Hazard ratios of total and cause specific mortality in *47,XYY *compared to age-matched males**. Time at risk was calculated from date of diagnosis until date of censoring (see Materials and Methods for details). The causes are divided in nineteen chapters according to the International Classification of Diseases. Only informative chapters are included.

Due to date of death in 2007 or later, the causes of deaths were not available in one *47,XYY *person and in 187 controls. For another ten controls the date of death was before 2007 and without known cause of death. These ten deaths were only included in the analysis of total mortality. Using Cox regression we identified a significantly increased total mortality, with a HR of 3.6 (2.6-5.1). The HRs of all informative chapters (corresponding to chapters with at least one deceased person and one deceased control) are shown in Figure 5.

## Discussion

Nationwide we have identified all males diagnosed with a diagnosis compatible with *47,XYY *and identified a significantly increased total mortality. Generally, cause specific mortality was increased compared to age and gender matched controls. Mortality data have to our knowledge not before been reported in a nationwide cohort with this specific karyotype. Our finding of a total mortality ratio of 3.6 is in comparison or even more pronounced than findings in Turner Syndrome [15,16] or Klinefelter Syndrome [17] or *47,XXX *[18] and also higher than found in the only other study in a *47,XYY *cohort by Swerdlow et al [14] with a relative risk of 1.9 (1.20-2.85), although with overlapping confidence interval. The reduction of lifespan of 10.3 years is even more than the expected loss of median lifespan in heavy smokers compared to non-smokers in Denmark [19]. In addition, we demonstrate a considerable delay in diagnosis and a low prevalence of *47,XYY*.

In all informative chapters cause specific mortality was generally increased. Analyzing the chapters separately, we identified a significantly increased mortality in cancer, neurological, and pulmonary diseases, trauma and the chapter of unspecified diseases. Further, we identified a statistically significant HR in the chapters concerning the skin, urological diseases, and chromosomal disorders. These analyses are compromised by a small number of deaths among the *47,XYY *persons (n = 1), and we consider the significant HR in these three chapters as a chance finding, however in line with the generally increased mortality. The British data showed a significantly increased mortality in pulmonary diseases only [14]. Time at risk is comparable in the British study (3 174 years) and the present one (3 373 years), but the distribution of age at diagnosis cannot readily be extracted from the former study. However, our exact matching of index-persons and controls is methodologically superior. If a similar approach was possible in the British study, probably further significant chapters would have been identified.

We have no obvious explanation for the finding of significantly increased mortality in the various ICD-10 chapters. However, it is noteworthy that the HR of the cardiovascular diseases reached 2.2 (0.99-5.04), p = 0.05, and was as such not significant. We presume that an increased number of index-persons or increased time of observation would have identified a significant HR here as well. It is important to note the total number of deaths in the chapters and not only the significance level. When few deaths among *47,XYY *persons are registered, estimates of hazard ratio have a wide confidence interval and hence are relatively imprecise. Due to the limited number of deaths, we have not undertaken analyses regarding eventual sub grouping in any of the chapters, apart from the trauma chapter. Due to the early reports regarding *47,XYY *males being overrepresented in prisons [20,21] we scrutinized data in the trauma chapter. Here, a total of five *47,XYY *persons and 176 controls died. The deaths in the *47,XYY *persons were due to suicide in an oligophrenic person (n = 1), traffic accident (a pedestrian addicted to drugs) (n = 1), various somatic lesions due to foreign body in the gastro-intestinal system (n = 1), due to overdose with tricyclic antidepressive (n = 1) and a subarachnoid hemorrhage whilst abusing heroin (n = 1). It is important to note that three of these deaths include either an overdose or drug abuse. We recommend that the possibility of a diagnosis of *47,XYY *is considered in drug addicted men who are tall [22,23] or have other stigmata.

The average prevalence of 14.1 per 100 000 is lower than the expected prevalence of 98 in 100 000, however higher than the finding by Abramsky et al in a smaller study from three English laboratories [11]. Here 5.3 percent of the estimated number of *47,XYY *persons were identified. In the study by Abramsky at al., six *47,XYY *persons were younger and five older than 20 years at diagnosis, which we consider comparable with our finding of a median age at diagnosis of 17.1 years. Only very large scale studies of huge populations will be sufficient to fully establish the exact prevalence of *47,XYY*.

The reduction of age at diagnosis during the study period may signal an increased awareness of *47,XYY *among physicians. Males identified in a clinical setting are not comparable to those identified in surveys, basically due to the presumed highly variable phenotype among *47,XYY *persons spanning from a normal phenotype to a clearly abnormal phenotype. Thus, males identified using our approach of focusing only on all persons diagnosed naturally bias the results. However, for the time being there is no other possible way of identifying the remaining undiagnosed males nationwide, and, more importantly, it is the persons diagnosed that we see in the daily clinic, or who themselves know that they have this chromosomal abnormality. It is important to emphasize that this report only includes males *diagnosed *with this karyotype. Thus, we consider this cohort as representative of the *47,XYY *males being seen by clinicians at the current point of time. To which degree the fact that only a limited percentage of the *47,XYY *persons are identified, influences the increased mortality is not known. However, we expect that inclusion of more *47,XYY *persons, possibly less stigmatized, will tend to reduce the increased mortality found in the present study.

In conclusion, in this first nationwide study in diagnosed *47,XYY *persons we have identified an average prevalence of 14.1 per 100 000, which is lower than the expected of 98 per 100 000. The *47,XYY *persons are diagnosed relatively late with a median age at diagnosis of 17.1 years. Their total mortality is significantly increased compared to age and gender matched controls from the background population. Furthermore, this increased mortality is present in all informative chapters according to the ICD-10 and significantly increased in the following: cancer, neurological, and pulmonary diseases, trauma and the chapter of unspecified diseases. Much more needs to be learned about this syndrome and clinical studies should be conducted in order to identify clinical problems enabling future decrease in the increased risk of death.

## Competing interests

The authors declare that they have no competing interests.

## Authors' contributions

KS made substantial contributions to conception and design, as well as analysis and interpretation of data and drafted the manuscript. SJ made contributions to conception and design, interpretation of data and revised it critically. CG made substantial contributions to conception and design and interpretation of data and revised it critically. All authors have given final approval of the version to be published.
